# Latent antibiotic resistance genes are abundant, diverse, and mobile in human, animal, and environmental microbiomes

**DOI:** 10.1186/s40168-023-01479-0

**Published:** 2023-03-08

**Authors:** Juan Salvador Inda-Díaz, David Lund, Marcos Parras-Moltó, Anna Johnning, Johan Bengtsson-Palme, Erik Kristiansson

**Affiliations:** 1 Department of Mathematical Sciences, Chalmers University of Technology and University of Gothenburg, SE-412 96 Gothenburg, Sweden; 2 Centre for Antibiotic Resistance Research in Gothenburg (CARe), Gothenburg, Sweden; 3 Department of Systems and Data Analysis, Fraunhofer-Chalmers Centre, Gothenburg, Sweden; 4 Division of Systems and Synthetic Biology, Department of Life Sciences, SciLifeLab, Chalmers University of Technology, Gothenburg, Sweden; 5 Department of Infectious Diseases, Institute of Biomedicine, The Sahlgrenska Academy, University of Gothenburg, Gothenburg, Sweden

**Keywords:** Antimicrobial resistance, Metagenomics, Emerging resistance genes, Pan-resistome, Core-resistome

## Abstract

**Background:**

Bacterial communities in humans, animals, and the external environment maintain a large collection of antibiotic resistance genes (ARGs). However, few of these ARGs are well-characterized and thus established in existing resistance gene databases. In contrast, the remaining latent ARGs are typically unknown and overlooked in most sequencing-based studies. Our view of the resistome and its diversity is therefore incomplete, which hampers our ability to assess risk for promotion and spread of yet undiscovered resistance determinants.

**Results:**

A reference database consisting of both established and latent ARGs (ARGs not present in current resistance gene repositories) was created. By analyzing more than 10,000 metagenomic samples, we showed that latent ARGs were more abundant and diverse than established ARGs in all studied environments, including the human- and animal-associated microbiomes. The pan-resistomes, i.e., all ARGs present in an environment, were heavily dominated by latent ARGs. In comparison, the core-resistome, i.e., ARGs that were commonly encountered, comprised both latent and established ARGs. We identified several latent ARGs shared between environments and/or present in human pathogens. Context analysis of these genes showed that they were located on mobile genetic elements, including conjugative elements. We, furthermore, identified that wastewater microbiomes had a surprisingly large pan- and core-resistome, which makes it a potentially high-risk environment for the mobilization and promotion of latent ARGs.

**Conclusions:**

Our results show that latent ARGs are ubiquitously present in all environments and constitute a diverse reservoir from which new resistance determinants can be recruited to pathogens. Several latent ARGs already had high mobile potential and were present in human pathogens, suggesting that they may constitute emerging threats to human health. We conclude that the full resistome—including both latent and established ARGs—needs to be considered to properly assess the risks associated with antibiotic selection pressures.

Video Abstract

**Supplementary Information:**

The online version contains supplementary material available at 10.1186/s40168-023-01479-0.

## Background

The increasing number of infections by antibiotic-resistant bacteria is a growing problem with almost 5 million associated yearly deaths worldwide [1]. Bacteria become resistant to antibiotics through changes in their DNA, often by acquiring antibiotic resistance genes (ARGs) through the process of horizontal gene transfer [2]. Many ARGs are located on mobile genetic elements (MGEs), such as transposons and conjugative elements (e.g., plasmids), which enable them to spread efficiently between cells, including from non-pathogenic and commensal bacterial species to pathogens [3]. Several thousand ARGs have been described to date, encoding resistance mechanisms to almost all clinically used antibiotics. This number is constantly increasing due to the influx and discovery of novel, often more efficient, resistance determinants [4–6]. The source of most ARGs, however, remains unknown which hampers the implementation of proper management strategies to stop this gene flow [7, 8].

Bacterial communities in humans, animals, and the external environment maintain large collections of ARGs [9–11]. Only a small number of these ARGs—typically those that have been encountered in clinical pathogens—are established, well-characterized, and thus available in existing resistance gene databases (denoted “established ARGs” in this study). The remaining latently present ARGs (denoted “latent ARGs”) are, in contrast, less, if at all, studied. Recently, computational methods have been used to systematically explore the latent ARGs present in bacterial genomes to describe their evolutionary relationship to the established ARGs. For example, the prediction of novel ARGs from bacterial sequence data expanded the number of known macrolide ARGs more than tenfold [12]. A plethora of latent ARGs has been described in other clinically relevant classes of ARGs, including genes conferring resistance to $\beta$-lactams, tetracyclines, and quinolones [13–18]. A wide range of latent ARGs has also been identified by functional metagenomics, and several studies describe a surprisingly diverse resistome in many bacterial communities, including the human microbiome [19–21]. However, little is known about how latent ARGs are distributed within and between environments.

Metagenomics, defined as the high-throughput sequencing of microbial samples, is commonly used to study bacterial communities, including the presence of resistance genes. Today’s rich literature provides detailed ARG abundance profiles in various environments, including human- and animal-associated microbiomes and a wide range of external environments such as marine and freshwater, soil, and wastewater [22–24]. However, existing studies have focused on ARGs available in reference databases such as ResFinder [25], CARD [26] or ARGs-OAP [27]. Even though these databases are comprehensive, they contain almost exclusively well-established genes already encountered in pathogens. Consequently, existing studies have greatly underestimated the abundance and diversity of ARGs in bacterial communities [28]. Clearly, data on the total resistome—including both latent and established ARGs—is necessary to understand how bacterial communities react and adapt to selection pressures from the anthropogenic use of antibiotics. Further insights into the abundance and diversity of latent ARGs are also essential to properly identify risk environments where resistance genes are most likely to be mobilized and, eventually, transferred into pathogens.

In this study, we aimed to provide a more complete view of the resistome. A large and diverse reference database of both latent and established ARGs was created by combining the ResFinder repository with computationally predicted resistance genes from half a million bacterial genomes. Analysis of more than 10, 000 metagenomic samples showed that latent ARGs were ubiquitously present in high abundance and diversity in all studied environments. The pan-resistomes, i.e., the set of all ARGs present in an environment, were heavily dominated by latent ARGs, while the core-resistomes, i.e., the genes that were commonly encountered in an environment, consisted of both established and latent ARGs. Several of the latent core-resistome ARGs were shared between the environments and were present in human pathogens. Analysis of the genomic context showed that a majority of these genes were associated with MGEs, including mechanisms for conjugation, suggesting that they may be emerging resistance determinants in pathogens. We conclude that both latent and established ARGs need to be considered to adequately describe the resistome and the effect of antibiotic selection pressures on bacterial communities.

## Methods

### Analysis of metagenomes

Metagenomes were retrieved from the MGnify database (2020-10-14) [29]. To ensure comparability, only data from the Illumina platforms were included. Also, samples were excluded if the study had less than five sequencing runs in total or if unique links to forward or reverse raw fastq files in the European Nucleotide Archive Portal API (ENA) [30] were missing. This resulted in 22,272 metagenomic samples where their raw fastq files, which were retrieved from the ENA repository, consisted of $6.3\times 10^{11}$ reads encompassing $7.3\times 10^{13}$ nucleotides (50TB of compressed data). BBDuk from BBMap version 38.87 [31] was used for quality control, with trim quality 20, minimum length 60, and left and right trimming of the raw files. Sequencing runs with at least 5 million reads remaining after the quality control were considered for analysis, adding up to 10,375 metagenomic samples (Additional file 1: Table S1).

### Computational prediction of ARGs

A total of 427,495 bacterial genomes consisting of 47,582,748 sequences (NCBI GenBank database, 2019-10-22) [32] were analyzed using fARGene (v0.1, default parameters). We used fARGene in this study since it has been shown to have a high performance and its predictions have been experimentally verified on several occasions [12, 13, 15, 16, 33, 34]. fARGene was executed using 17 hidden Markov model gene profiles for ARGs conferring resistance to five major classes of antibiotics: for $\beta$-lactams, we defined gene classes A, B1/B2, B3, and D [13, 33]; for aminoglycosides, gene classes $aac(2')$, *aac*(3), $aac(6')$, $aph(2^{\prime \prime })$, $aph(3')$, and *aph*(6); for macrolides, gene classes *erm* and *mph* [12]; for quinolones, gene class *qnr* [16]; and for tetracyclines, gene classes efflux pumps, inactivating enzymes (monooxygenases), and ribosomal protection genes (RPGs) [15] (downloaded from https://github.com/fannyhb/fargene). All matches satisfying the previously reported model-specific significance thresholds for full-length genes were considered to be putative ARGs and stored for further analysis [12, 13, 16, 33, 35].

### Creation of the ARG reference database

A reference database of antibiotic resistance genes was created consisting of two collections of genes. The first collection comprised 2466 resistance gene sequences present in the ResFinder repository [25] (accessed 2019-10-01) that were correctly classified by at least one of the fARGene models. ResFinder was used since it consists of well-established mobile ARGs. The second collection consisted of 74,904 unique sequences of putative resistance genes from the fARGene analysis. As open reading frames of insertion sequence transposases could overlap with ARGs, we used BLASTx 2.2.31 [36] with default parameters to align the resistance gene sequences to 7057 transposases from the ISFinder database (accessed 2022-02-08) [37, 38]. Gene sequences with at least 80% identity level and at least 20 amino acid overlap to any transposase were removed from the data set (120 sequences removed).

To reduce redundancy, all gene sequences were clustered at a nucleotide cut-off of 90% using VSEARCH version 2.7.0 [39]. The resulting 23,367 centroids were used as representative sequences for each cluster and are hereafter referred to as ARGs. Next, BLASTp version 2.2.31 [36] with default parameters was used to align all ARGs to all ResFinder sequences. The ARGs with an identity level of at least 90% and with a match overlap of at least 70% to any sequence in ResFinder were labeled as “established ARGs.” This cut-off is commonly used for assigning reads to resistance genes and, thus, established ARGs will primarily consist of genes typically analyzed in shotgun metagenomic studies [40]. The ARGs with an identity level below 90% or with a match overlap shorter than 20% were labeled as “latent” ARGs. The ARGs not fulfilling any of these criteria were removed (three sequences removed). In total, we labeled 588 ARGs as established and 22,504 ARGs as latent.

### Quantification of ARGs

Latent and established ARGs were quantified by aligning metagenomic forward reads to the reference databases using DIAMOND blastx version 2.0.4 [41]. A strict identity threshold of 95% was employed, with a read coverage of at least 20% and a minimum match length of 20 amino acids. For reads that matched multiple ARGs, the match with firstly the highest identity, secondly the longest alignment, or thirdly the lowest *e*-value was considered. When there was a tie in identity, match length, and *e*-value, one of the ARGs was randomly selected. To avoid double-counting, only the forward read in each read pair was used.

The relative abundance $C_{ij}$ for gene class *i* in metagenome *j* was transformed and normalized according to $C_{ij}=1000\sqrt{\frac{r_{ij}}{T_j}}$, where $r_{ij}$ is the number of reads matching any ARG from class *i* in the metagenomic sample *j* and $T_j$ is the total number of metagenomic reads in sample *j*. The $\alpha$-diversity was calculated as richness, i.e., the number of unique ARGs identified within a sample. When calculating the $\alpha$-diversity, all samples were rarefied to 5,000,000 reads. The abundance and diversity were calculated for each environment and each class of ARGs, both in total and separately for latent and established ARGs. The principal component analysis (PCA) was done using log-transformed relative abundances.

### Estimation of the pan- and core-resistome

The pan- and core-resistomes were calculated for each environment by randomly taking 1000 subsamples of size 100 metagenomic samples from the rarefied metagenomes. For each subsample, we stored (1) the unique number of ARGs present in any sample ($\alpha$-diversity) for the pan-resistome calculations and (2) the ARGs present in at least 50% of the samples (commonly encountered gene set) for the core-resistome calculation. The size of the pan-resistome for each environment was calculated as the average $\alpha$-diversity over the 1000 subsamples, and the core-resistome as the number of ARGs present in at least 900 of the commonly encountered gene sets. For the environments having less than 100 samples (lentic water, rhizosphere, and respiratory system), all samples were used to compute the pan- and core-resistomes.

### Genetic context analysis

The genetic context analysis was done in the host genomes of the latent genes in the core-resistomes. The analysis included 1429 unique resistance gene sequences corresponding to 136 ARGs. For each ARG, the closest known homolog was identified using BLASTx v2.10.1 [36] to align the gene sequences against the CARD database [26]. Here, CARD was used since it is more comprehensive than ResFinder and includes, in contrast to ResFinder, some genes that are not clinically relevant and/or mobile. Then, genetic regions of up to 10,000 base pairs upstream and downstream of the gene sequences were retrieved using GEnView v0.1.1 [42] and screened for the presence of genes associated with MGEs and integrons. The genetic regions were translated in all six reading frames using EMBOSS Transeq v6.5.7.0 [43] and searched with 124 HMMs from MacSyfinder Conjscan v2.0 representing genes involved in conjugation [44], using HMMER v3.1b2 [45]. Insertion sequences (ISs) and other mobile ARGs were identified by applying BLASTx v2.10.1 [36]. For IS elements, a reference database based on ISFinder [37, 38] was used to find the best among overlapping hits, with the alignment criteria that hits should display $>50\%$ coverage and $>90\%$ amino acid identity to a known IS transposase, as well as being located within 1,000 base pairs of the latent resistance gene (upstream or downstream). For co-localized mobile ARGs, ResFinder v4.0 was used as a reference database [25], with the alignment criterion that hits should display an amino acid identity $>90\%$ to a known ARG. Finally, the genetic regions were searched for integrons using Integron Finder v1.5.1 [46]. After the screening, the genetic contexts were manually investigated and curated.

## Results

In this study, we analyzed the abundance, diversity, and environmental spread of latent and established ARGs from 17 major resistance gene classes. An ARG reference database was created from the large-scale screening of almost 500,000 publicly available bacterial whole-genome assemblies using fARGene, a method that uses optimized gene models to identify putative resistance genes [33], and resistance genes reported in ResFinder, a database with well-characterized mobile clinically relevant ARGs [25]. After clustering the gene sequences, ARGs were classified as “established” if they showed high sequence similarity with any gene reported in ResFinder and “latent” if they were sufficiently dissimilar (see the “Methods” section for full details). The final database consisted of 23,092 ARGs, of which 99%, 97%, 94%, 93%, and 96% were classified as latent aminoglycoside, $\beta$-lactam, macrolide, quinolone, and tetracycline resistance genes, respectively (Table 1).

**Table 1:** Analyzed and detected antibiotic resistance genes with their relative abundance and $\alpha$-diversity

		Number of	Detected	Abundance	$\alpha$-diversity
		ARGs	% of ARGs	(median)	(median)
**Aminoglycosides**					
$aac(2')$	L	594	58%	0	0
	E	6	83%	0	0
*aac*(3)	L	2121	65%	0.49	1
	E	27	89%	0	0
$aac(6')$	L	1861	60%	0.68	2
	E	51	100%	0	0
$aph(2^{\prime \prime })$	L	306	42%	0	0
	E	6	100%	0	0
$aph(3')$	L	1144	69%	0.53	1
	E	26	81%	0.69	1
*aph*(6)	L	2551	70%	0	0
	E	8	88%	0	0
$\beta$-**lactams**					
A	L	5038	74%	1.72	11
	E	105	95%	1.47	5
B1/B2	L	1054	56%	0	0
	E	55	98%	0	0
B3	L	1921	86%	0.71	3
	E	14	86%	0	0
C	L	1224	93%	0.28	0
	E	45	100%	0	0
D	L	1771	77%	0	0
	E	93	96%	0	0
**Macrolides**					
*erm*	L	554	59%	0.60	2
	E	46	87%	1.39	6
*mph*	L	430	55%	0	0
	E	19	100%	0	0
**Quinolones**					
*qnr*	L	273	68%	0	0
	E	20	95%	0	0
**Tetracyclines**					
Efflux	L	381	96%	0	0
	E	34	100%	0.22	0
Enzyme	L	250	95%	0	0
	E	12	100%	0	0
Ribosomal protection	L	1031	98%	1.36	10
	E	21	100%	2.62	14

The latent (L) and established (E) ARGs are listed per type of antibiotic and gene class, with the number of ARGs in the reference database and the proportion of the ARGs detected in at least one metagenomic sample. The relative abundance and $\alpha$-diversity correspond to the median of the log-scale abundance and $\alpha$-diversity over all metagenomes. The detected percentage of ARGs corresponds to the number of genes that had a match in any of the metagenomic fragments analyzed divided by the total number of genes

The abundance of the ARGs was analyzed in 10,375 metagenomic samples from 20 types of environments, including, human- and animal-associated, marine and freshwater, soil, and wastewater microbiomes (Additional file 1: Table S1). The samples contained a total of 1.98$\times 10^{11}$ reads, of which 0.06% were matched to at least one ARG, and 2.29$\times 10^{13}$ base pairs. The proportion of ARGs detected in at least one metagenome differed substantially between gene classes: from $\sim$100% for latent and established tetracyclines resistance genes, down to 55% for latent macrolide resistance genes (Table 1).

### Latent and established ARGs showed distinct abundance and diversity patterns

**Fig. 1:**
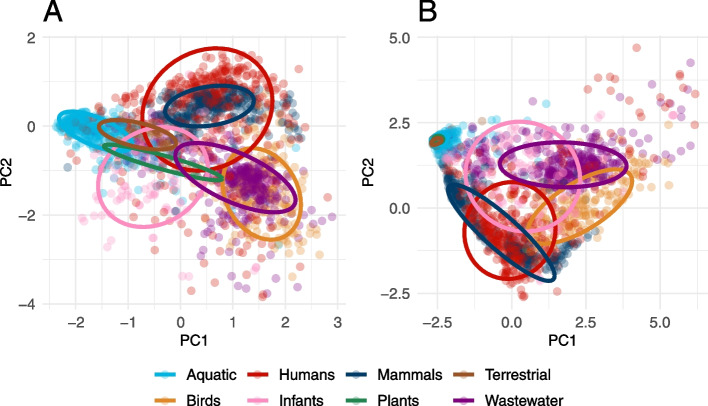
Principal component analysis of the abundance of **A** latent and **B** established ARGs. Aquatic includes samples from fresh, lentic, and marine water; Plants includes rhizosphere samples; Infants include samples from their digestive system; Wastewater includes activated sludge, water and sludge, fecal source, and raw wastewater; Terrestrial includes soil samples; Human includes samples from the skin, digestive and respiratory systems; Birds includes samples from their digestive system; and Mammals includes samples from the digestive systems of bovines, mice, and pigs. The analysis was based on log-transformed ARG abundance using all metagenomic samples but a maximum of 400 per environment are shown. The ellipses are calculated based on a multivariate t-distribution at a 75% confidence level. Latent ARGs were in general more diverse than established ARGs and showed less overlap especially for the aquatic, terrestrial, and plant environments

Principal component analysis (PCA) of the relative gene abundances showed different patterns for latent and established ARGs. The abundance of the latent ARGs formed distinct clusters that showed separation between the environments (Fig. 1A). There was, however, an overlap between humans, mammals, and, to some extent, also infants and wastewater, demonstrating similarities in their ARG abundance profiles. In contrast, the established ARGs had less variable clusters, especially for several of the external environments, reflecting their relatively low abundance of ARGs (Fig. 1B). Moreover, the established ARGs showed, contrary to the latent ARGs, no overlap between the host-associated metagenomes and the external environments (excluding wastewater). The wastewater and bird digestive systems resistome formed diverse clusters that, for latent ARGs, partially overlapped.

**Fig. 2:**
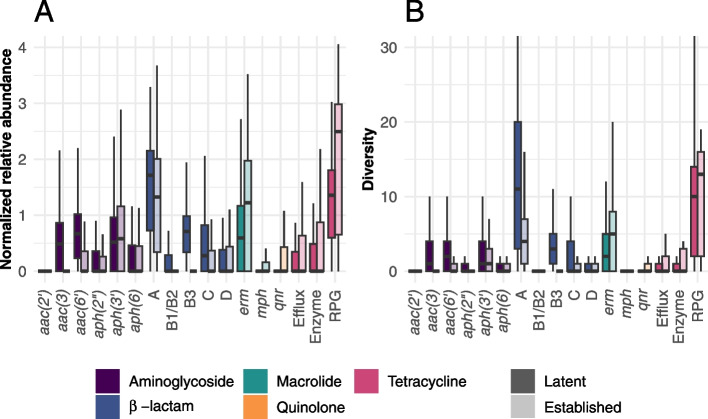
Distribution of the **A** relative abundance and **B** $\alpha$-diversity for latent and established ARGs. Distribution of the log-transformed abundance and $\alpha$-diversity per gene class. The colors indicate antibiotic type with higher opacity for latent ARGs and higher transparency for established ARGs. RPG is short for ribosomal protection gene

Analysis of individual gene classes showed that most latent aminoglycosides and $\beta$-lactam ARGs were generally more abundant and diverse than the established ARGs (Fig. 2; *p*$<1\times 10^{-12}$ in Additional File 1: Table S2). The difference in diversity was especially pronounced for $\beta$-lactam resistance genes where latent class A $\beta$-lactamases had a twofold higher diversity (difference in median over all samples), a trend that was consistent across several environments (Additional File 2: Figs. S2-S3). Interestingly, the macrolide and tetracycline resistance genes showed contrasting results, where the established ARGs were more abundant and diverse compared to latent ARGs, *p*$=1\times 10^{-10}$ (Additional File 1: Table S2). This was especially emphasized in the host-associated and wastewater metagenomes (Additional File 2: Figs. S2-S3). A similar pattern could also be seen for quinolone resistance genes, for which mainly established ARGs were detected, primarily in infants, birds, and wastewater (Additional File 2: Figs. S2-S3).

### Latent ARGs are more abundant than established ARGs in most environments

**Fig. 3:**
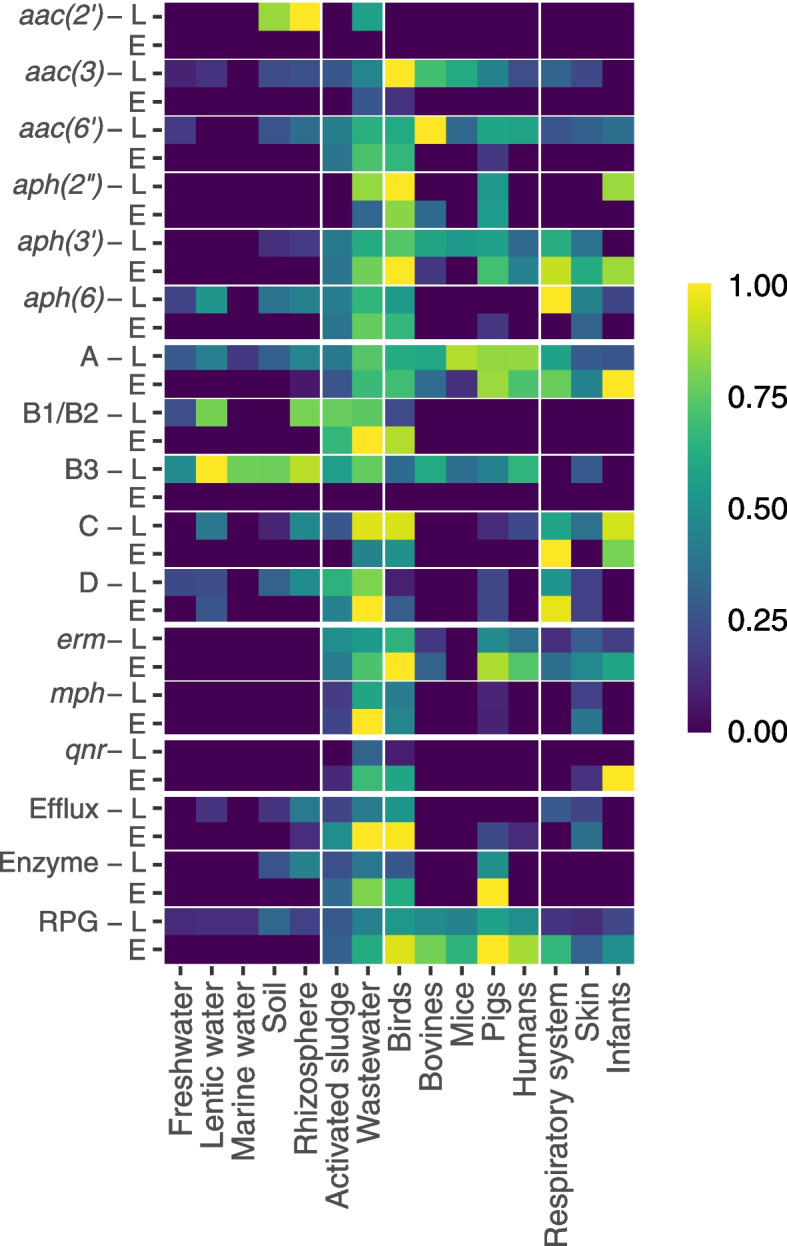
Relative abundance of latent and established ARGs divided per gene class and environment. Each gene class is represented by two rows: L for latent and E for established. The labels Birds, Bovines, Mice, Pigs, Humans, and Infants denote metagenomes from the corresponding digestive system. Respiratory system and skin only include human samples. The color intensity reflects the gene- and environment-specific relative abundance, which was calculated based on the median of the relative log-transform abundance over all samples from the environment. To make the genes comparable, all values were normalized based on the environment with the highest abundance. RPG is short for ribosomal protection gene

Analysis of the gene abundances across the environments showed that latent and established ARGs were distributed differently (Fig. 3). Latent genes from several classes were widely present in the analyzed environments, including aminoglycoside resistance genes *aac*(3), $aac(6')$, $aph(3')$, class A and B3 $\beta$-lactamases, and tetracycline ribosomal protection genes (RPGs). For established genes, this was only true for class A $\beta$-lactamases. Furthermore, latent ARGs were more abundant than established ARGs in all external environments except for wastewater, especially aminoglycoside, $\beta$-lactam, and tetracycline resistance genes. The human- and mammal-associated metagenomes contained a high abundance of latent and/or established ARGs where $aac(6')$, class A $\beta$-lactamases, *erm* genes, and RPGs were almost ubiquitously present. Additionally, there were strong similarities between the distributions of latent and established ARGs in human, pig, and bovine digestive system resistomes (especially pronounced for *aac*(3), $aac(6')$, $aph(3')$, class A and B3 $\beta$-lactamases, *erm* genes, and RPGs). Wastewater and birds had the highest abundance of both latent and established ARGs where only established $aac(2')$ and class B3 $\beta$-lactamases were rare (Additional File 2: Figs. S2-S3). Interestingly, only one gene class, latent $aac(2')$, was only found in the external environments but not in any of the host-associated metagenomes.

There was a positive correlation between latent and established genes (Additional File 2: Fig. S4), which was strong for *aph*(6) and tetracycline efflux pumps, especially in digestives systems, activated sludge, and wastewater ($0.67< \rho < 0.99$, p$< 1\times 10^{-6}$). Much lower correlations were seen for class B3 $\beta$-lactamases and $aac(6')$, which were only significant in the human skin and digestive system, respectively.

### Analysis of the pan-resistomes and core-resistomes reveals commonly encountered latent ARGs

**Fig. 4:**
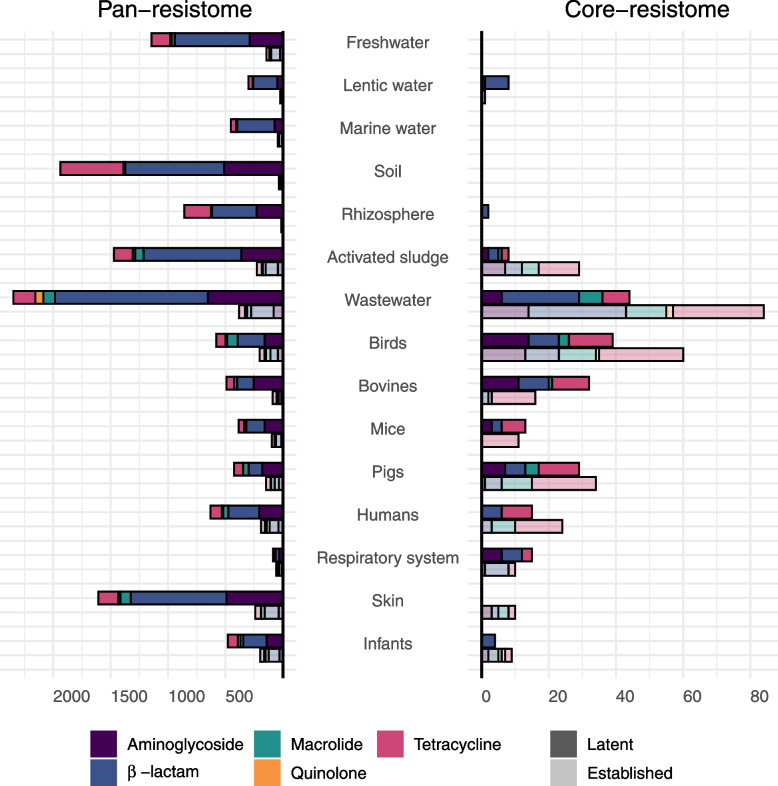
Pan-resistomes and core-resistomes. The length of the left and right bars describe the size of the pan- and core-resistome, respectively. The pan-resistome includes all genes encountered in at least one sample from the environment, and the core-resistome includes all genes that were commonly encountered (at least 50% of the samples). The colors indicate antibiotic type with higher opacity for latent ARGs and higher transparency for established ARGs. The computations were done based on rarefied metagenomes that, for each environment, were repeatedly subsampled down to 100 samples. The figure shows the average number of genes over all 100 samples. The labels Birds, Bovines, Mice, Pigs, Humans, and Infants denote metagenomes from the corresponding digestive system. Respiratory system and skin only include human samples

Next, we analyzed the size and diversity of the latent and established resistomes by calculating their corresponding pan-resistome. This was done for each environment by repeatedly sampling 100 metagenomes and counting the number of genes that were detected in at least one of the samples (Fig. 4; see the “Methods” section). The size of the pan-resistomes varied considerably between the environments. The metagenomes from the external environments (soil, wastewater, activated sludge) and human skin had the largest pan-resistomes with, on average, $>1700$ ARGs. In contrast, the number of ARGs in the pan-resistomes of the digestive systems was more than twofold lower ($<800$ genes on average). All pan-resistomes were dominated by latent ARGs, a pattern that was especially pronounced in the external environments where 86% to 98% were latent ARGs. In comparison, 74% to 85% of the pan-resistome ARGs were latent in the digestive systems of humans, mammals, and birds.

To investigate if specific classes of ARGs were selected for within an environment, the pan-resistomes were compared to their corresponding core-resistome, defined as the ARGs that were commonly encountered in an environment (detected in at least 50% of the randomly sampled metagenomes; see the “Methods” section). The sizes of the core-resistomes were, as expected, considerably smaller than the pan-resistomes—on average, the core-resistome accounted for 4.7% of the matching pan-resistome (Fig. 4). The external environments except wastewater had small core-resistomes with less than ten genes on average, suggesting that they contain few ARGs that are ubiquitously present over the detection limit. In contrast, the core-resistomes for the digestive systems (excluding infants) were larger, ranging between 24 and 99 ARGs, constituting between 5% and 13% of their corresponding pan-resistomes. In the human microbiome, the digestive system had the largest core-resistome (39 ARGs, 4.8% of the pan-resistome) followed by the respiratory tract (25 ARGs, 17.2%) and skin (10 ARGs, 0.5%). The wastewater metagenomes had the overall largest core-resistome, containing as many as 128 genes (4.7% of the pan-resistome), suggesting that a significant collection of ARGs is commonly encountered in wastewater treatment plants.

Moreover, the latent ARGs constituted a large part of the core-resistomes. In the host-associated metagenomes, the proportion of latent ARGs varied between 31% (infants) to 67% (bovine). The core-resistome of wastewater contained the largest number of latent ARGs (44 ARGs), corresponding to 34% of the total core-resistome. Although the pan-resistome of activated sludge was relatively large (62% the size of the wastewater pan-resistome), the core-resistome was considerably smaller (29% the size of the wastewater).

We, furthermore, noted that the distribution of ARG classes differed between the core- and pan-resistomes (Additional File 1: Table S3, S4). Tetracycline resistance genes were, for example, overrepresented in the core- compared to the pan-resistomes in the human, bird, and mammal digestive systems (*p*$=1\times 10^{-6}$), while aminoglycoside resistance genes were underrepresented in humans and pigs (*p*$=1\times 10^{-4}$). This pattern could be seen for both latent and established ARGs (Fig. 4). Indeed, for the human digestive system, as many as 59% of the core-resistome ARGs were tetracycline resistance genes of which 64% were latent. In contrast, not a single aminoglycoside resistance gene, neither latent nor established, was identified to be part of the human digestive system core-resistome.

### Latent ARGs were shared between host-associated environments

**Fig. 5:**
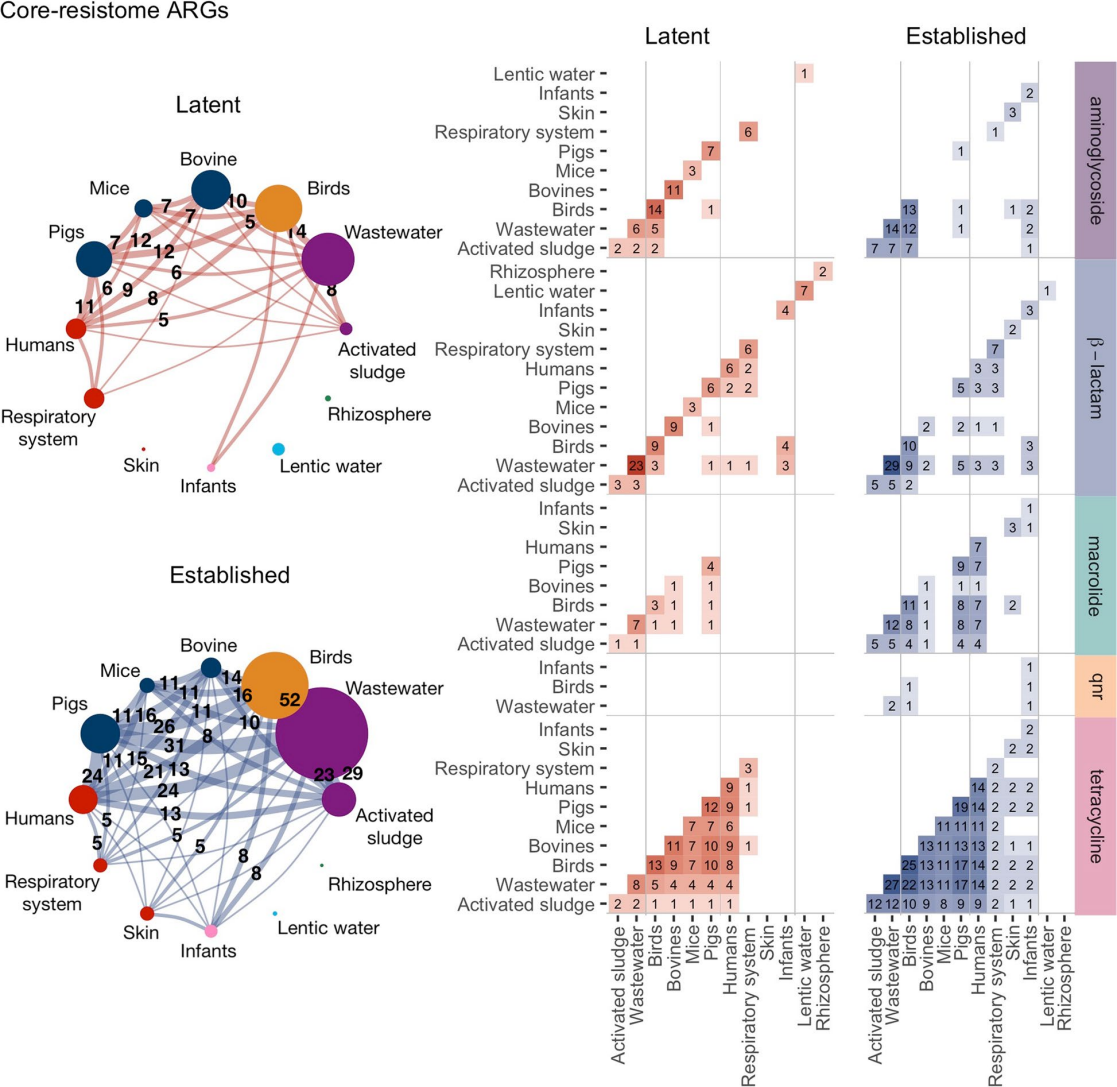
Core-resistome overlap between environments. **a** Networks where nodes represent environments with their node size proportional to the size of the environment’s core-resistome. The width of the edge is proportional to the number of core-resistome ARGs shared between two environments. Only overlaps of at least five ARGs are shown. **b** The heatmaps show the number of core-resistome ARGs shared between two environments for each antibiotic type. The labels Birds, Bovines, Mice, Pigs, Humans, and Infants denote metagenomes from the corresponding digestive system. Respiratory system and skin only include human samples. Qnr is an abbreviation for quinolone resistance

Established ARGs are known to spread between environments, including human and animal microbiomes. To investigate if this was also true for latent ARGs, we visualized the overlap between the core-resistomes from different environments as networks (Fig. 5). Remarkably, all host-associated metagenomes, as well as wastewater and activated sludge, contained ARGs that were shared between their core-resistomes. For the human digestive system, 73% of the latent and 100% of the established ARGs in the core-resistome were also encountered in at least one other environment. The largest number of genes shared with the human resistome was found in the pig digestive system (11 latent and 24 established ARGs, 90% of the core-resistome), followed by birds (8 latent and 21 established ARGs, 74%), and wastewater (5 latent and 24 established ARGs, 72%). In contrast, the overlap between the human digestive system and the core-resistomes from other parts of the human microbiome (skin and respiratory system) was substantially lower. Finally, the largest number of overlapping ARGs between any environments was seen between the wastewater and bird metagenomes (14 latent and 52 established). The shared part of the core-resistome consisted mainly of tetracycline resistance genes. Latent macrolide, $\beta$-lactam, and aminoglycoside resistance genes were also shared mainly between birds and wastewater but also between the digestive systems of other species. All latent and established ARGs found in activated sludge were also found in the core-resistome of wastewater.

### Context analysis shows indications of mobility for several commonly encountered latent ARGs

The established ARGs used in this study are mobile and can thus efficiently spread between bacteria. To investigate the potential for horizontal transfer of the latent core-resistome ARGs, we annotated their genomic context in their host bacteria for (1) genes related to mobile genetic elements (MGEs) commonly associated with ARGs (conjugative elements, transposons, and integrons) and (2) co-localized established ARGs (see the “Methods” section). Of the 108 latent ARGs present only in core-resistomes of a single environment, 15.7% were located with an MGE-associated gene, while 7.4% were located next to an established ARG (Table 2 and Additional File 3: Table S6). For the 29 latent ARGs commonly encountered in at least two environments, these proportions increased to 48% and 21%, respectively. Finally, 31 (23%) of the latent ARGs present in the core-resistome were found in at least one human pathogenic species.

**Table 2:** Context analysis of selected latent core-resistome ARGs

Cluster ID$^1$	Closest ARG$^2$	Host	Host$^3$	Associated	Co-localized	Environments$^4$
ARG class	[% identity]	phyla	species	MGE/integron	ARGs	
**Tetracyclines**						
C8 RPG	Tet(W/N/W)	Act,	*Collinsella* sp.,	MPF$_{\textrm{FATA}}$, MOB$_{\textrm{Q}}$	*erm*(B)	Birds, Bovine,
[12]	[80%]	Firmicutes	*LActinobacteriaobacillus* sp.			Humans, Mice,
						Pigs, Wastewater
C7 RPG	Tet(44)	Pro,	*Campylobacter fetus [P]*	MPF$_{\textrm{FATA}}$,		Birds, Bovine,
[2]	[86%]	Firmicutes		MOB$_{\textrm{Q}}$, MOB$_{\textrm{P1}}$		Humans, Mice, Pigs
C10 RPG	Tet(32)	Firmicutes	*Clostridium clostridioforme*,	MPF$_{\textrm{FA}}$, MOB$_{\textrm{P}1}$		Birds, Bovine,
[7]	[76%]		*Dorea formicigenerans*,			Mice, Pigs
C14 RPG	Tet(32)	Firmicutes	*Acetatifactor muris*,	MPF$_{\textrm{FATA}}$, MOB$_{\textrm{Q}}$,		Birds, Bovine,
[3]	[88%]		*Blautia coccoides*	MOB$_{\textrm{P1}}$		Humans, Mice, Pigs
$\beta$-**lactams**						
C34 A	CepA-44	Bacteroidetes	*Bacteroides vulgatus [P]*,			Humans
[31]	[54%]		*Bacteroides massiliensis [P]*			
C46 A	cepA	Bacteroidetes	*Bacteroides caecimuris*,	MPF$_{\textrm{FA}}$,		Mice
[11]	[41%]		*Parabacteroides distasonis*	MPF$_{\textrm{FATA}}$		
C35 A	CfxA3	Bacteroidetes	*Bacteroides fragilis [P]*,	MPF$_{\textrm{B}}$		Humans, Pigs,
[8]	[84%]		*Bacteroides eggerthii*			Respiratory system
C37 A	CfxA6	Bacteroidetes	*Bacteroides vulgatus [P]*,	MOB$_{\textrm{B}}$		Pigs
[3]	[81%]		*Bacteroides ovatus*			
C30 C	ACC-7	Proteobacteria	*Thauera humireducens*,	Class 1 integrase,	*tet*(G)	Activated
[3]	[47%]		*Thauera* sp.	IS91-family transposase		sludge, Wastewater
C22 C	ADC-221	Proteobacteria	*Acinetobacter baumannii [P]*,	MOB$_{\textrm{Q}}$	*bla*$_{\mathrm {OXA-58}}$	Wastewater
[6]	[61%]		*Acinetobacter bereziniae*			
C89 D	OXA-209	Bacteroidetes	*Myroides odoratimimus [P]*	MPF$_{\textrm{B}}$	*bla*$_{\mathrm {OXA-347}}$,	Wastewater
[1]	[89%]				*tet*(X)	
C94 D	OXA-209	Bacteroidetes	*Vaginella massiliensis*		*tet*(X4)	Wastewater
[1]	[73%]					
**Macrolides**						
C50 *erm*	Erm(42)	Firmicutes,	*Anaerofustis stercorihominis*,	MPF$_{\textrm{FA}}$		Birds, Bovine,
[6]	[48%]	Proteobacteria	*Dechloromonas aromatica*,			Pigs, Wastewater
C41 *erm*	Erm(42)	Firmicutes,	*Salmonella enterica [P]*,	MPF$_{\textrm{FATA}}$,		Birds
[4]	[42%]	Proteobacteria	*Eubacterium rectale*	MOB$_{\textrm{Q}}$, MOB$_{\textrm{P1}}$		
C105 *erm*	Erm(F)	Bacteroidetes	*Bacteroides fragilis [P]*,	MPF$_{\textrm{B}}$, MOB$_{\textrm{P1}}$	*tet*(Q)	Wastewater
[3]	[63%]		*Alistipes onderdonkii*			
C38 *erm*	Erm(42)	Firmicutes	*Solobacterium* sp.	MPF$_{\textrm{FA}}$, MPF$_{\textrm{FATA}}$,		Pigs
[5]	[49%]			MOB$_{\textrm{V}}$		
C109 *erm*	Erm(X)	Actinobacteria	*Corynebacterium dentalis*,	MOB$_{\textrm{F}}$		Birds
[2]	[57%]		*Trueperella pyogenes*			
C67 *mph*	Mph(E)	Bacteroidetes	*Myroides odoratimimus [P]*	MOB$_{\textrm{P1}}$		Wastewater
[1]	[83%]					
**Aminoglycosides**						
C87 *aac(3)*	AAC(3’)-IXa	Firmicutes	*Lactobacillus amylovorus*,	MOB$_{\textrm{T}}$		Birds, Pigs
[13]	[30%]		*Lactobacillus gallinarum*			
C132 *aac(6’)*	AAC(6’)-Iad	Firmicutes	*Clostridioides difficile [P]*	MPF$_{\textrm{FATA}}$,		Pigs
[2]	[56%]			MOB$_{\textrm{Q}}$		
C130 *aac(6’)*	AAC(6’)-Im	Firmicutes	*Ruminococcus flavefaciens*,	MPF$_{\textrm{FATA}}$		Bovine
[14]	[55%]		*Butyrivibrio* sp.			
C128 *aac(6’)*	AAC(6’)-Ib7	Proteobacteria	*Pseudomonas aeruginosa [P]*	Class 1 integron	*aac(6’)*-31, *aadA6*,	Birds,
[1]	[74%]				*sul1*, *bla*$_{\mathrm {OXA-2}}$	Wastewater
C84 *aph(3’)*	APH($3^{\prime \prime }$)-Ib	Proteobacteria	*Vibrio cholerae [P]*		*aph(6)-Id*	Birds, Activated
[1]	[89%]					sludge, Wastewater

Gene sequences of the 1429 variants (clustered into 133 ARGs) of all latent core-resistome ARGs are found in Additional File 3: Table S5. The corresponding information for all latent core-resistome ARGs is found in Additional File 3: Table S6.$^{1}$Cluster ID and ARG class with the number of gene sequences in the cluster given in brackets.$^{2}$The closest established ARG identified by protein alignment against CARD with the identity level given in brackets. $^{3}$Pathogenic species are indicated with [P].$^{4}$Environments where the ARG was considered part of the core-resistome.

## Discussion

In this study, we investigated the abundance and diversity of latent ARGs, which are largely uncharacterized and only sporadically represented in the current databases. From the analysis of a wide range of bacterial communities, represented by more than 10,000 metagenomic samples, we showed that latent ARGs are, compared to established ARGs, both more abundant and diverse. This pattern was especially pronounced for the soil and aquatic bacterial communities. More surprisingly was that wastewater, the human and animal digestive systems—all known to contain a large collection of established resistance genes [9, 22]—also harbored a high diversity of latent ARGs. Furthermore, several of the latent ARGs commonly encountered in either wastewater or the host-associated environments were also shared between environments, especially between humans, pigs, birds, and wastewater. We also showed that these widespread latent ARGs were, to a large extent, mobilized and associated with integrons, conjugative elements, and insertion sequences. Additionally, several of the commonly encountered latent ARGs were found in species from multiple phyla and several pathogens, including *Pseudomonas aeruginosa*, *Salmonella enterica*, and *Campylobacter spp*. This study thus demonstrates that there are latent ARGs enriched in the human and animal microbiomes that are already both mobile and present in virulent pathogens (including ESKAPE) [47]. This suggests that some latent ARGs may constitute major risks to human health [8, 48, 49].

Our analysis showed that there were similarities in the distribution of latent and established ARGs between metagenomes. The correlation between the abundance of latent and established ARGs was overall positive but, for most gene classes, moderate in size, which is in accordance with earlier results based on a smaller set of latent ARGs [28]. However, stronger correlations were found for macrolide and tetracycline resistance genes in the host-associated and wastewater metagenomes. Moreover, latent and established ARGs showed similar over- and under-representation patterns in the core-resistomes. In particular, both latent and established tetracycline and macrolide resistance genes were overrepresented in the core-resistomes compared to their pan-resistomes. In contrast, $\beta$-lactamases and aminoglycoside resistance genes were instead underrepresented. It is plausible that the similar patterns of latent and established ARG abundance observed in this study are a result of antibiotic consumption. Tetracyclines and macrolides are antibiotics that are efficient in the anaerobic conditions where these bacterial communities thrive [50]. Other antibiotics, such as aminoglycosides are, in comparison, generally less efficient under anaerobic conditions [51], and may not induce as strong selection pressures in the digestive system, which may reduce the relative abundance of the corresponding ARGs. Nevertheless, our results suggest that latent and established ARGs are, at least partially, affected by similar selection pressures. Consideration of the larger resistome—including the large diversity of latent ARGs—is, therefore, necessary to understand how antibiotic selection pressures affect bacterial communities and the selection of antibiotic resistance genes.

Several of the latent commonly encountered ARGs were shared between environments, especially the host-associated digestive systems. However, in relation to the composition of ARGs in our reference database, latent ARGs were, compared to established ARGs, shared to a less extent. Indeed, our results showed that most latent core-resistome ARGs are not widespread, at least not at detectable levels. This suggests that are strong evolutionary barriers that prevent many of the latent ARGs from horizontally transferring between hosts and efficiently spreading between environments [52]. Indeed, many of the latent ARGs were found in bacterial chromosomes and are, thus, most likely not as mobile as the established ARGs, which are often located on mobile genetic elements known to efficiently spread to pathogens [53]. There are also fitness costs associated with the horizontal acquisition of genes, which may require latent ARGs to adapt to their new host before they provide a significant evolutionary advantage [54]. Indeed, many types of ARGs require a high expression to induce a significant resistance phenotype and, therefore, codon optimization can be needed for efficient translation. Nevertheless, the continuous discovery of novel and more efficient ARGs in clinical setting suggest that the latent resistome still contains highly potent resistance determinants [55–57]. It is, therefore, possible that some of the common and shared latent ARGs identified in this study constitute emerging resistance genes, but are associated with either a high fitness cost or are located on inefficient mobile genetic elements which prevent them from spreading *en masse* among human pathogens.

The pan-resistomes were heavily dominated by latent ARGs. In the external environments, as much as 95% of the pan-resistome consisted of latent ARGs, while the number was somewhat lower in the host-associated environments ($>74\%$). Our results thus suggest that all environments contain large reservoirs of latent ARGs and can serve as important sources of new resistance determinants. Indeed, new ARGs are constantly being discovered in clinical settings, but their origins are often unknown [7]. This suggests that some resistance genes are likely transferred from bacteria that are not yet available in our sequence repositories. Considering that most of the human pathogens and commensals today have sequenced genomes, many ARGs are, therefore, likely to originate from non-clinical bacteria [4, 8]. Indeed, the majority of the origins of 37 investigated established ARGs came from bacterial species commonly found in the environment, but which were interestingly also sporadically associated with the human microbiome [7]. This indicates that antibiotic selection pressure is likely a factor promoting the mobilization and successive transfer of latent ARGs into pathogens [52]. Our results show that, in addition to the human microbiome, wastewater-associated bacterial communities may be high-risk environments for the mobilization of new clinically relevant ARGs. Firstly, significant concentrations of antibiotics are commonly encountered in the influent of wastewater treatment plants, suggesting that the selection pressures needed to promote the mobilization of latent ARGs may be present [58–60]. Secondly, the wastewater metagenomes also contain established ARGs, many of which originate from the human microbiome, along with a plethora of MGEs that can efficiently transfer genes into a wide range of pathogens [61, 62]. Finally, as shown in this study, wastewater metagenomes also maintain a diverse pan-resistome with ARGs from all included gene classes. In fact, the wastewater pan-resistome was larger than in soil, reflecting an increased likelihood that compatible and efficient resistance determinants are present. Developing improved methods for the identification and surveillance of environments with an increased risk for mobilization of resistance genes will be critical to stopping the transfer of new efficient resistance determinants into human pathogens. Here, the characterization of both the latent and established resistome—and thus the full resistance potential of a bacterial community—will be crucial.

Shotgun metagenomics offers a holistic approach to studying bacterial communities. Commonly used databases, such as ResFinder, CARD, and ARGs-OAP [25–27], are highly focused on established ARGs already associated with clinical isolates and will, therefore, overlook latent ARGs and underestimate the total diversity of the resistome. Since no comprehensive reference database was available when this study was performed, putative ARGs were computationally predicted by analyzing almost half a million bacterial genomes. We used fARGene, a method that uses probabilistic gene models that are optimized both for sensitivity and specificity, where the latter is especially important to avoid false positives [33]. To make sure that our predictions are as accurate as possible, we limited the study to gene classes that had been thoroughly evaluated. Indeed, predictions from fARGene have been experimentally validated repeatedly and showed that between 70% and 86% of the predicted ARGs induce a resistance phenotype when expressed in *Escherichia coli* [12, 13, 15, 16, 34]. This suggests that a large proportion of our latent ARGs are likely to be correct and thus provide resistance in at least some bacterial species. Thus, the reference database assembled for this study could be applied in other areas where broad screening of ARGs are important, such as diagnostics and surveillance of antibiotic-resistant bacteria. We, furthermore, argue that larger and more diverse reference databases are essential for the analysis of ARGs using shotgun metagenomics. Indeed, our study shows that 38% of the ARGs commonly encountered in the human digestive system are latent and there is a risk that these genes are overlooked in analyses based on existing databases. However, even if computational methods can be used to explore latent ARGs, the available bacterial genomic and metagenomic sequencing data only represent a tiny fraction of the total microbial diversity on earth [63]. The abundance and diversity of latent ARGs presented here should thus be considered conservative estimates. The latent resistome is, consequently, likely to be substantially larger than what is depicted in this study.

## Conclusion

This study constitutes the first large-scale analysis of the latent resistome, i.e., the collection of antibiotic resistance genes present in bacterial communities that are not (yet) considered major clinical problems and is, therefore, largely uncharacterized. Our findings show that latent ARGs are ubiquitously present in relatively high abundance in all analyzed environments and have a diversity that surpasses that of established resistance genes. Many commonly encountered latent ARGs are associated with MGEs and detected at high abundance in multiple environments. More alarmingly, several MGE-associated latent ARGs are already present in highly virulent human pathogens. This suggests that they may provide an evolutionary advantage and reduce the efficacy of antibiotic treatments in the clinic. We conclude that both latent and established ARGs need to be considered to capture the consequences of antibiotic selection pressures and how they affect the promotion and spread of ARGs. The origin of the vast majority of clinically relevant ARGs—including those that have been recently described—remains unknown. It is therefore vital that the diversity and characteristics of the latent resistome are included as a component in the assessment of risk environments for the mobilization of new ARGs. This may facilitate the implementation of improved management strategies that limit the introduction of new resistance determinants in human pathogens and clinical settings.

## Supplementary information

**Additional file 1: Table S1** shows the number of metagenomic samples per environment included in the study. **Table S2** shows the results for the assessment of higher and lower abundance and diversity of the established ARGs compared to the latent ones for each antibiotic class. **Table S3** shows the results for the assessment of over- and under-representation of ARGs in the core-resistomes compared to their corresponding pan-resistome for each antibiotic type. **Table S4** shows the number of ARGs in the pan- and core-resistomes for each environment and gene class.

**Additional file 2: Figure S1 **shows the principal component analysis (PCA) of the *α*-diversity of latent and established ARGs. Figures 2 and 3 correspond to the distribution of the log-transformed relative abundance and *α*-diversity of latent and established ARGs for each gene class and environment. **Figure S2** shows the host-associated metagenomes and **Figure S3** the external environments. Aquatic includes samples from fresh, lentic, and marine water; Plants includes rhizosphere samples; Infants include samples from their digestive system; Wastewater includes activated sludge, water and sludge, fecal source, and raw wastewater; Terrestrial includes soil samples; Human includes samples from the skin, digestive and respiratory systems; Birds includes samples from their digestive system; and Mammals includes samples from the digestive systems of bovines, mice, and pigs. RPG is short for ribosomal protection gene. Figure 4 shows the correlation between the abundance of latent and establish ARGs for each gene class and environment. The color intensity reflects the size of the estimated correlation coefficient and an asterisk (*) marks significant correlations (*p*<0.001). Gray squares indicate environment and gene classes with an insufficient number of non-zero observations to calculate the correlation coefficient and/or the *p*-value.

**Additional file 3: Table S5** shows all latent core-resistome ARGs and the fARGene gene sequences within their clusters (90% identity level). The table includes the gene sequence identifiers; the clusters to which each sequence belongs; which of the sequences were cluster centroids and, hence, used as the representative sequence for the ARG; and the nucleotide sequences. **Table S6** shows the context analysis of all latent core-resistome ARGs. The table includes the cluster identifiers; ARG class; the number of gene sequences in the cluster; the closest established ARG with % amino acid identity to CARD; host phyla of the gene sequences when identified; host species of the gene sequences when identified with human pathogens marked; genes associated with mobile genetic elements (MGEs) identified in the genetic context of the gene; co-localized ARGs in the genetic context of the gene; and the environments where the ARG was considered part of the core-resistome. **Table S7** corresponds to the 10,375 metagenomic run IDs included in this study and retrieved from the ENA. **Table S8** corresponds to the NCBI Assembly IDs used to build the fARGene database.

## Data Availability

The data analyzed in this study consisted of pre-existing datasets retrieved from public repositories. Full details are specified in Methods.
