# Supplementary material for: Latent antibiotic resistance genes are abundant, diverse, and mobile in human, animal, and environmental microbiomes
Source: Microbiome. 2023 Mar 8;11:44. doi: 10.1186/s40168-023-01479-0 (PMC9993715; doi:10.1186/s40168-023-01479-0)
Supplement: Supplementary file 2 — Additional file 1: Table S1 shows the number of metagenomic samples per environment included in the study. Table S2 shows the results for the assessment of higher and lower abundance and diversity of the established ARGs compared to the latent ones for each antibiotic class. Table S3 shows the results for the assessment of over- and under-representation of ARGs in the core-resistomes compared to their corresponding pan-resistome for each antibiotic type. Table S4 shows the number of ARGs in the pan- and core-resistomes for each environment and gene class. [file 40168_2023_1479_MOESM1_ESM.pdf]

Table S1: Number of metagenomes per environment. We used a three level classification for each environment. The number of metagenomic samples correspond to the number of runs in MGnify.

**External environments**			**Metagenomic samples**
Aquatic		Freshwater	112
		Lentic	45
		Marine	1,819
Terrestrial		Soil	200
Plants		Rhizosphere	15
Wastewater	Processed	Activated Sludge	104
	Raw	Water and sludge	125
		Fecal source	20
		Other	167
**Host-associated environments**			**Runs**
Birds		Digestive system	208
Mammals	Bovines	Digestive system	159
	Mice	Digestive system	492
	Pigs	Digestive system	811
Human		Digestive system	5,747
		Respiratory system	15
		Skin	235
Infant		Digestive system	101

Table S2: Comparison of the distribution of the abundance and diversity of established and latent ARGs. Established ARGs have a significantly lower ("-"), or higher ("+"), abundance or diversity compared to latent ARGs. Wilcoxon signed-rank tests were used to compare the log-scale abundance and α-diversity of established and latent ARGs over all metagenomes, only significant tests are reported ($p<1 \times 10^{-6}$).

Class	Data	"-/+"	W-statistic	p-value
aac(2')	Abundance	-	10536	2.2×10^{-16}
	Diversity	-	1422.5	2.2×10^{-16}
aac(3)	Abundance	-	2398138	2.2×10^{-16}
	Diversity	-	1484392	2.2×10^{-16}
aac(6')	Abundance	-	6509587	2.2×10^{-16}
	Diversity	-	3939519	2.2×10^{-16}
aph(2")	Abundance	-	6738596	2.2×10^{-16}
	Diversity	-	4288486	2.3×10^{-13}
aph(3')	Abundance	+	24491048	2.2×10^{-16}
	Diversity	-	13929091	2.2×10^{-16}
Class A	Abundance	-	21095855	2.2×10^{-16}
	Diversity	-	7764696	2.2×10^{-16}
Class B1/B2	Diversity	-	5121998	2.2×10^{-16}
Class B3	Abundance	-	33038	2.2×10^{-16}
	Diversity	-	30406	2.2×10^{-16}
Class C	Abundance	-	7541506	2.2×10^{-16}
	Diversity	-	4399004	2.2×10^{-16}
Class D	Abundance	+	11574444	2.8×10^{-16}
	Diversity	+	6916569	2.1×10^{-13}
erm	Abundance	+	33047241	2.2×10^{-16}
	Diversity	+	25752996	2.2×10^{-16}
mph	Abundance	+	4898584	2.1×10^{-16}
	Diversity	+	2862980	2.2×10^{-16}
qnr	Abundance	+	9220933	2.2×10^{-16}
	Diversity	+	4784812	2.2×10^{-16}
Efflux	Abundance	+	15846516	2.2×10^{-16}
	Diversity	+	10764721	2.2×10^{-16}
Enzyme	Abundance	+	14280048	2.2×10^{-16}
	Diversity	+	10517835	2.2×10^{-16}
RPG	Abundance	+	39604395	2.2×10^{-16}
	Diversity	+	27758043	2.2×10^{-16}

Table S3: Assessment of over- and under-representation of gene classes in the core-resistome compared to the pan-resistome. Classes are significantly under- ("-") and over-represented ("+") in the core-resistome in comparison to the pan-resistome. Fisher's exact test was used to compare the proportion of each antibiotic type and only significant values ($p=1\times10^{-3}$) are reported.

Biome	Antibiotic	"-/+"	Odds ratio	p-value
Activated sludge	β-lactams	-	0.21	2.1×10^{-5}
	Tetracyclines	+	4.4	7.7×10^{-5}
Birds	β-lactams	-	0.38	8.6×10^{-5}
	Tetracyclines	+	3.4	2.5×10^{-7}
Bovines	Tetracyclines	+	4.79	8.1×10^{-7}
Humans	Aminoglycosides	-	0	1.4×10^{-6}
	Tetracyclines	+	7.4	5.8×10^{-9}
Mice	Tetracyclines	+	17.16	3.1×10^{-10}
Pigs	Aminoglycosides	-	0.24	2.6×10^{-5}
	Tetracyclines	+	3.73	2.0×10^{-6}
Wastewater	β-lactams	-	0.54	4.4×10^{-4}
	Macrolides	+	3.35	3.5×10^{-5}
	Tetracyclines	+	3.86	3.9×10^{-9}

Table S4: Number of ARGs in the pan- and core-resistomes for each environment and gene class. The labels Birds, Bovines, Mice, Pigs, Humans, and Infants denotes metagenomes from the corresponding digestive system. Respiratory system and skin only include human samples. The number in parenthesis correspond to the ARGs in the pan-resistome. RPG is short for ribosomal protection gene.

	Aminoglycosides					
	aac(2')	*aac(3)*	*aac(6')*	*aph(2")*	*aph(3')*	*aph(6)*
Activated sludge	0 (8)<br>0 (0)	0 (103)<br>0 (8)	0 (85)<br>2 (14)	0 (6)<br>0 (3)	1 (55)<br>2 (15)	1 (107)<br>3 (4)
Birds	0 (1)<br>0 (0)	6 (40)<br>0 (6)	2 (43)<br>2 (15)	1 (4)<br>0 (6)	4 (46)<br>8 (13)	1 (26)<br>3 (4)
Bovines	0 (1)<br>0 (0)	8 (98)<br>0 (0)	1 (70)<br>0 (2)	0 (5)<br>0 (2)	2 (63)<br>0 (9)	0 (18)<br>0 (3)
Freshwater	0 (34)<br>0 (0)	0 (69)<br>0 (0)	0 (57)<br>0 (13)	0 (20)<br>0 (0)	0 (39)<br>0 (10)	0 (71)<br>0 (3)
Humans	0 (0)<br>0 (0)	0 (65)<br>0 (4)	0 (72)<br>0 (12)	0 (6)<br>0 (6)	0 (45)<br>0 (13)	0 (18)<br>0 (3)
Infants	0 (0)<br>0 (0)	0 (36)<br>0 (4)	0 (21)<br>0 (10)	0 (4)<br>0 (2)	0 (27)<br>2 (11)	0 (54)<br>0 (3)
Lentic water	0 (0)<br>0 (0)	0 (10)<br>0 (0)	0 (9)<br>0 (2)	0 (0)<br>0 (0)	0 (7)<br>0 (1)	1 (23)<br>0 (0)
Marine water	0 (1)<br>0 (0)	0 (22)<br>0 (1)	0 (16)<br>0 (2)	0 (4)<br>0 (0)	0 (9)<br>0 (5)	0 (21)<br>0 (1)
Mice	0 (1)<br>0 (0)	3 (48)<br>0 (1)	0 (56)<br>0 (6)	0 (2)<br>0 (0)	0 (41)<br>0 (6)	0 (11)<br>0 (2)
Pigs	0 (0)<br>0 (0)	2 (72)<br>0 (3)	2 (62)<br>0 (11)	0 (4)<br>0 (5)	3 (39)<br>1 (11)	0 (4)<br>0 (4)
Respiratory system	0 (0)<br>0 (0)	1 (9)<br>0 (0)	0 (3)<br>0 (2)	0 (1)<br>0 (2)	1 (6)<br>1 (6)	4 (13)<br>0 (0)
Rhizosphere	0 (6)<br>0 (0)	0 (45)<br>0 (3)	0 (37)<br>0 (0)	0 (0)<br>0 (0)	0 (26)<br>0 (0)	0 (114)<br>0 (1)
Skin	0 (31)<br>0 (1)	0 (89)<br>0 (6)	0 (55)<br>0 (10)	0 (21)<br>0 (2)	0 (107)<br>3 (15)	0 (190)<br>0 (4)
Soil	0 (45)<br>0 (0)	0 (127)<br>0 (2)	0 (77)<br>0 (3)	0 (5)<br>0 (0)	0 (36)<br>0 (5)	0 (221)<br>0 (2)
Wastewater	0 (16)<br>0 (1)	1 (157)<br>0 (17)	1 (135)<br>4 (34)	1 (10)<br>0 (6)	1 (140)<br>7 (17)	2 (195)<br>3 (6)

Continued

Table S4 Continued.

	β-lactams				
	A	B1-B2	B3	C	D
Activated sludge	1 (304)<br>1 (39)	0 (53)<br>1 (10)	0 (138)<br>0 (1)	1 (127)<br>0 (21)	1 (229)<br>3 (37)
Birds	4 (157)<br>8 (32)	0 (6)<br>1 (6)	0 (26)<br>0 (0)	5 (34)<br>1 (14)	0 (11)<br>0 (13)
Bovines	9 (50)<br>2 (14)	0 (2)<br>0 (1)	0 (44)<br>0 (0)	0 (32)<br>0 (0)	0 (16)<br>0 (6)
Freshwater	0 (277)<br>0 (20)	0 (30)<br>0 (6)	0 (163)<br>0 (2)	0 (85)<br>0 (28)	0 (97)<br>0 (21)
Humans	6 (158)<br>3 (37)	0 (4)<br>0 (3)	0 (60)<br>0 (1)	0 (41)<br>0 (25)	0 (6)<br>0 (11)
Infants	0 (84)<br>2 (40)	0 (1)<br>0 (1)	0 (8)<br>0 (0)	4 (102)<br>1 (33)	0 (9)<br>0 (21)
Lentic water	3 (56)<br>0 (3)	1 (6)<br>0 (0)	2 (85)<br>0 (1)	1 (37)<br>0 (0)	0 (26)<br>1 (13)
Marine water	0 (91)<br>0 (12)	0 (23)<br>0 (1)	0 (131)<br>0 (0)	0 (31)<br>0 (3)	0 (53)<br>0 (9)
Mice	3 (108)<br>0 (21)	0 (2)<br>0 (3)	0 (25)<br>0 (2)	0 (19)<br>0 (16)	0 (7)<br>0 (4)
Pigs	6 (67)<br>5 (21)	0 (1)<br>0 (2)	0 (27)<br>0 (0)	0 (13)<br>0 (3)	0 (9)<br>0 (13)
Respiratory system	2 (9)<br>3 (11)	0 (0)<br>0 (0)	0 (3)<br>0 (0)	3 (17)<br>1 (3)	1 (3)<br>3 (10)
Rhizosphere	1 (140)<br>0 (4)	0 (22)<br>0 (1)	1 (117)<br>0 (1)	0 (48)<br>0 (0)	0 (62)<br>0 (1)
Skin	0 (343)<br>2 (41)	0 (35)<br>0 (10)	0 (135)<br>0 (6)	0 (233)<br>0 (32)	0 (89)<br>0 (33)
Soil	0 (318)<br>0 (6)	0 (22)<br>0 (1)	0 (308)<br>0 (1)	0 (62)<br>0 (1)	0 (145)<br>0 (6)
Wastewater	9 (530)<br>16 (67)	0 (66)<br>1 (21)	0 (215)<br>0 (6)	7 (293)<br>1 (42)	7 (228)<br>11 (60)

Continued

Table S4 Continued.

	Macrolides		Quinolones	Tetracyclines		
	erm	*mph*	*qnr*	Efflux	Enzyme	RPG
Activated sludge	1 (56)<br>4 (17)	0 (16)<br>1 (8)	0 (22)<br>0 (9)	0 (44)<br>3 (21)	0 (5)<br>0 (3)	2 (115)<br>9 (19)
Birds	3 (57)<br>9 (25)	0 (34)<br>2 (13)	0 (15)<br>1 (15)	2 (18)<br>8 (19)	0 (4)<br>1 (3)	11 (58)<br>16 (19)
Bovines	1 (13)<br>1 (14)	0 (14)<br>0 (4)	0 (0)<br>0 (0)	0 (6)<br>0 (13)	0 (3)<br>0 (3)	11 (56)<br>13 (19)
Freshwater	0 (16)<br>0 (9)	0 (12)<br>0 (2)	0 (14)<br>0 (4)	0 (31)<br>0 (10)	0 (13)<br>0 (5)	0 (117)<br>0 (12)
Humans	0 (41)<br>7 (18)	0 (4)<br>0 (8)	0 (11)<br>0 (13)	0 (18)<br>0 (13)	0 (21)<br>0 (3)	9 (60)<br>14 (19)
Infants	0 (23)<br>1 (19)	0 (1)<br>0 (8)	0 (23)<br>1 (11)	0 (16)<br>0 (14)	0 (12)<br>0 (3)	0 (60)<br>2 (18)
Lentic water	0 (1)<br>0 (2)	0 (1)<br>0 (0)	0 (2)<br>0 (0)	0 (15)<br>0 (1)	0 (1)<br>0 (0)	0 (21)<br>0 (0)
Marine water	0 (2)<br>0 (2)	0 (1)<br>0 (1)	0 (6)<br>0 (1)	0 (8)<br>0 (4)	0 (1)<br>0 (0)	0 (36)<br>0 (2)
Mice	0 (12)<br>0 (7)	0 (6)<br>0 (1)	0 (2)<br>0 (2)	0 (2)<br>0 (4)	0 (8)<br>0 (3)	7 (36)<br>11 (18)
Pigs	4 (33)<br>9 (20)	0 (15)<br>0 (8)	0 (2)<br>0 (5)	0 (10)<br>0 (19)	1 (6)<br>3 (3)	11 (61)<br>16 (19)
Respiratory system	0 (7)<br>0 (8)	0 (0)<br>0 (0)	0 (0)<br>0 (0)	2 (5)<br>0 (2)	0 (0)<br>0 (0)	1 (10)<br>2 (15)
Rhizosphere	0 (6)<br>0 (0)	0 (1)<br>0 (0)	0 (3)<br>0 (1)	0 (75)<br>0 (2)	0 (20)<br>0 (1)	0 (137)<br>0 (0)
Skin	0 (58)<br>2 (23)	0 (31)<br>1 (7)	0 (19)<br>0 (3)	0 (57)<br>0 (27)	0 (15)<br>0 (3)	0 (101)<br>2 (18)
Soil	0 (10)<br>0 (1)	0 (6)<br>0 (0)	0 (1)<br>0 (0)	0 (74)<br>0 (3)	0 (54)<br>0 (3)	0 (420)<br>0 (1)
Wastewater	3 (64)<br>8 (24)	4 (37)<br>4 (11)	0 (71)<br>2 (19)	1 (91)<br>9 (29)	0 (12)<br>2 (4)	7 (86)<br>16 (19)
