# Supplementary material for: Latent antibiotic resistance genes are abundant, diverse, and mobile in human, animal, and environmental microbiomes
Source: Microbiome. 2023 Mar 8;11:44. doi: 10.1186/s40168-023-01479-0 (PMC9993715; doi:10.1186/s40168-023-01479-0)
Supplement: Supplementary file 3 — Additional file 2: Figure S1 shows the principal component analysis (PCA) of the α-diversity of latent and established ARGs. Figures 2 and 3 correspond to the distribution of the log-transformed relative abundance and α-diversity of latent and established ARGs for each gene class and environment. Figure S2 shows the host-associated metagenomes and Figure S3 the external environments. Aquatic includes samples from fresh, lentic, and marine water; Plants includes rhizosphere samples; Infants include samples from their digestive system; Wastewater includes activated sludge, water and sludge, fecal source, and raw wastewater; Terrestrial includes soil samples; Human includes samples from the skin, digestive and respiratory systems; Birds includes samples from their digestive system; and Mammals includes samples from the digestive systems of bovines, mice, and pigs. RPG is short for ribosomal protection gene. Figure 4 shows the correlation between the abundance of latent and establish ARGs for each gene class and environment. The color intensity reflects the size of the estimated correlation coefficient and an asterisk (*) marks significant correlations (p<0.001). Gray squares indicate environment and gene classes with an insufficient number of non-zero observations to calculate the correlation coefficient and/or the p-value. [file 40168_2023_1479_MOESM2_ESM.pdf]

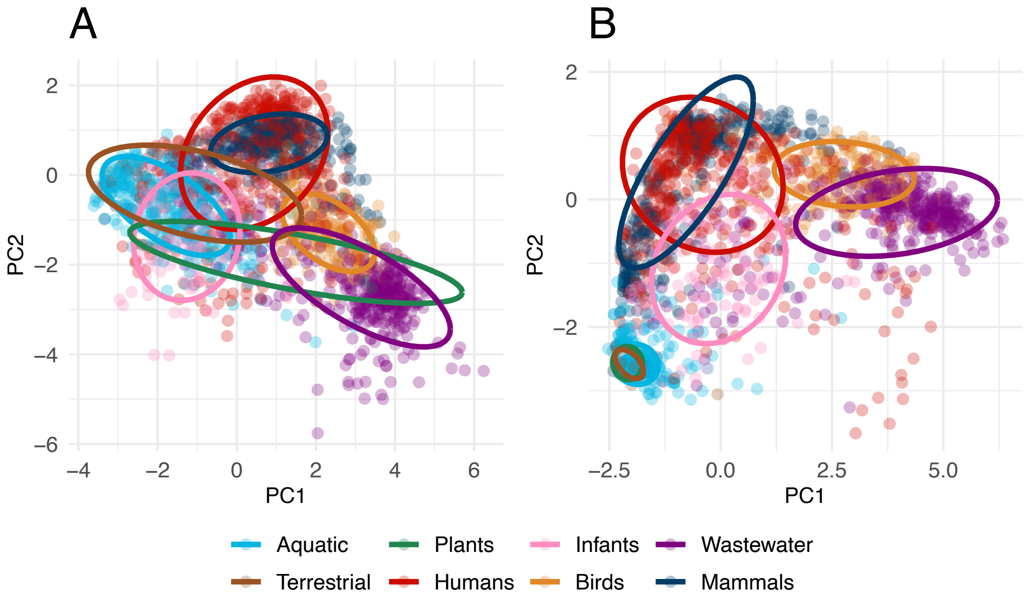

Figure S1: Principal component analysis (PCA) of the α-diversity of A) latent and B) established ARGs. Aquatic includes samples from fresh, lentic, and marine water; Wastewater includes activated sludge, water and sludge, fecal source, and raw wastewater; Birds includes samples from their digestive system; Mammals includes samples from the digestive systems of bovines, mice, and pigs; Human includes samples from the skin, digestive and respiratory systems; and Infants include samples from their digestive system. The PCA was done using rarefied gene counts of all the metagenomic samples, but a maximum of 400 samples per environment are shown. The ellipses are drawn from a multivariate t-distribution at a 75% confidence level. The aquatic, terrestrial, and plant environments are more widely spread for the latent ARGs, while they are gathered together for the established ARGs. In both PCAs, the human/mammal metagenomes form a cluster, while birds and wastewater are separated from the other environments.

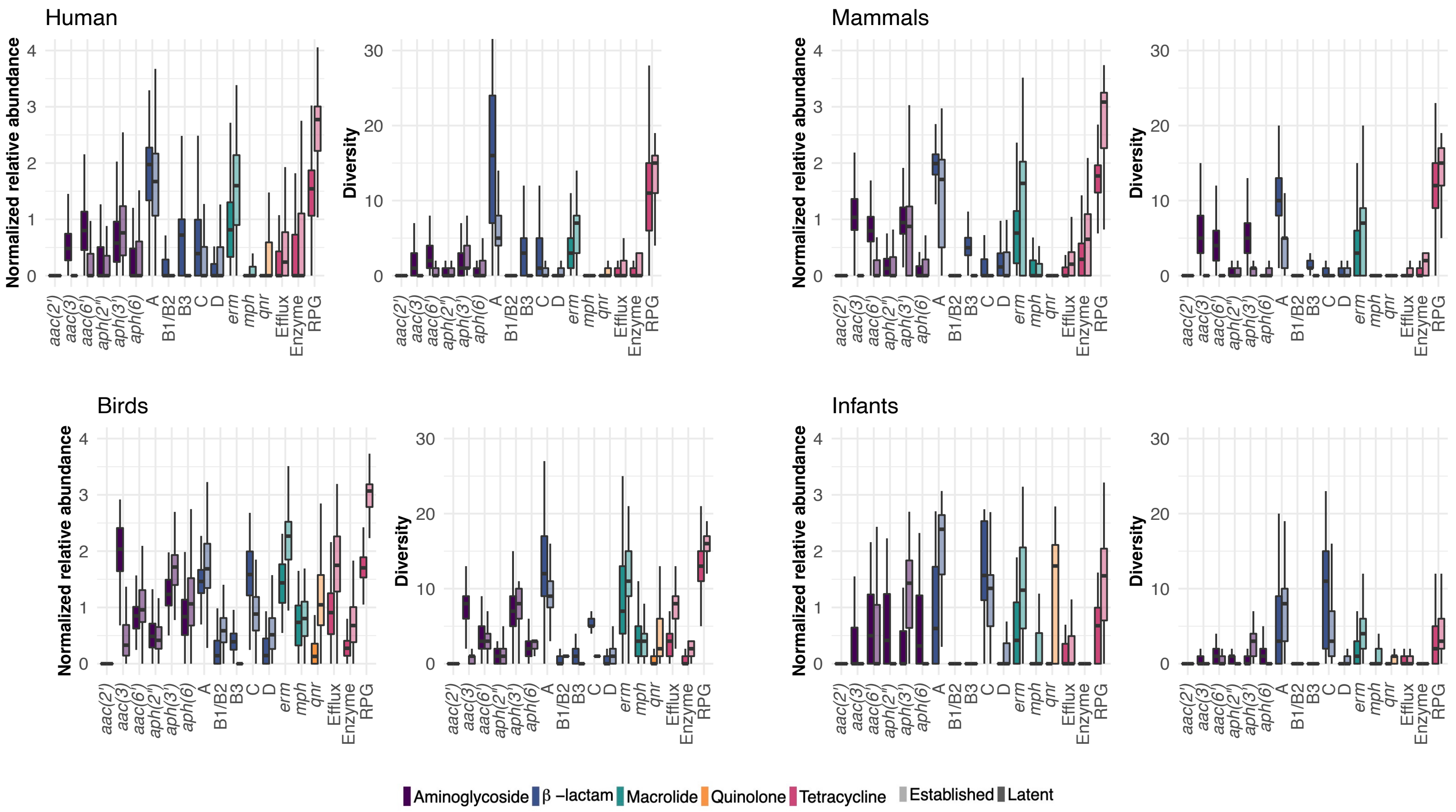

Figure S2: Distribution of the log-transformed abundance and α-diversity for latent and established ARGs for each gene class and environment. RPG is short for ribosomal protection gene.

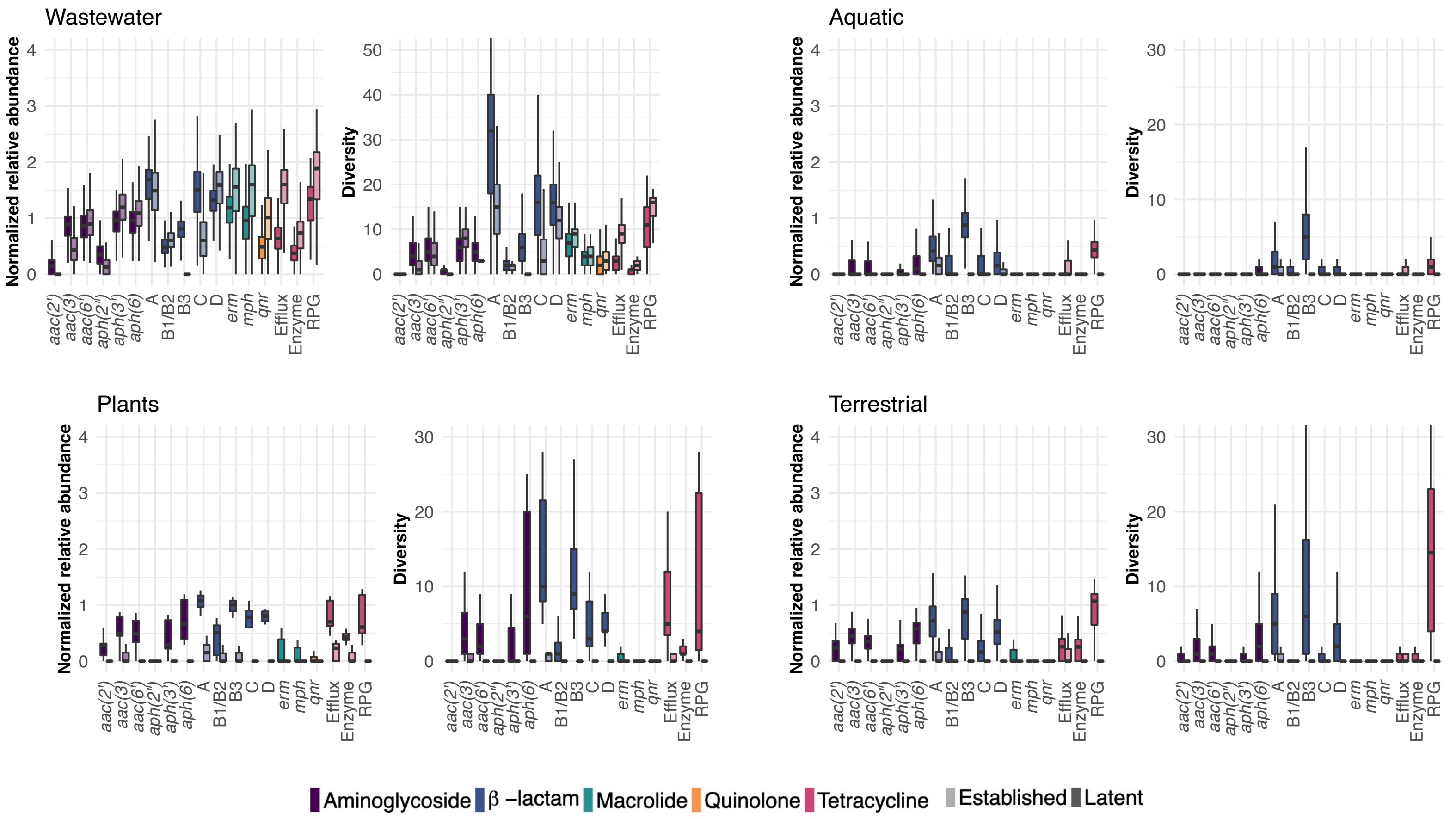

Figure S3: Distribution of the log-transformed abundance and α-diversity for latent and established ARGs for each gene class and environment. RPG is short for ribosomal protection gene.

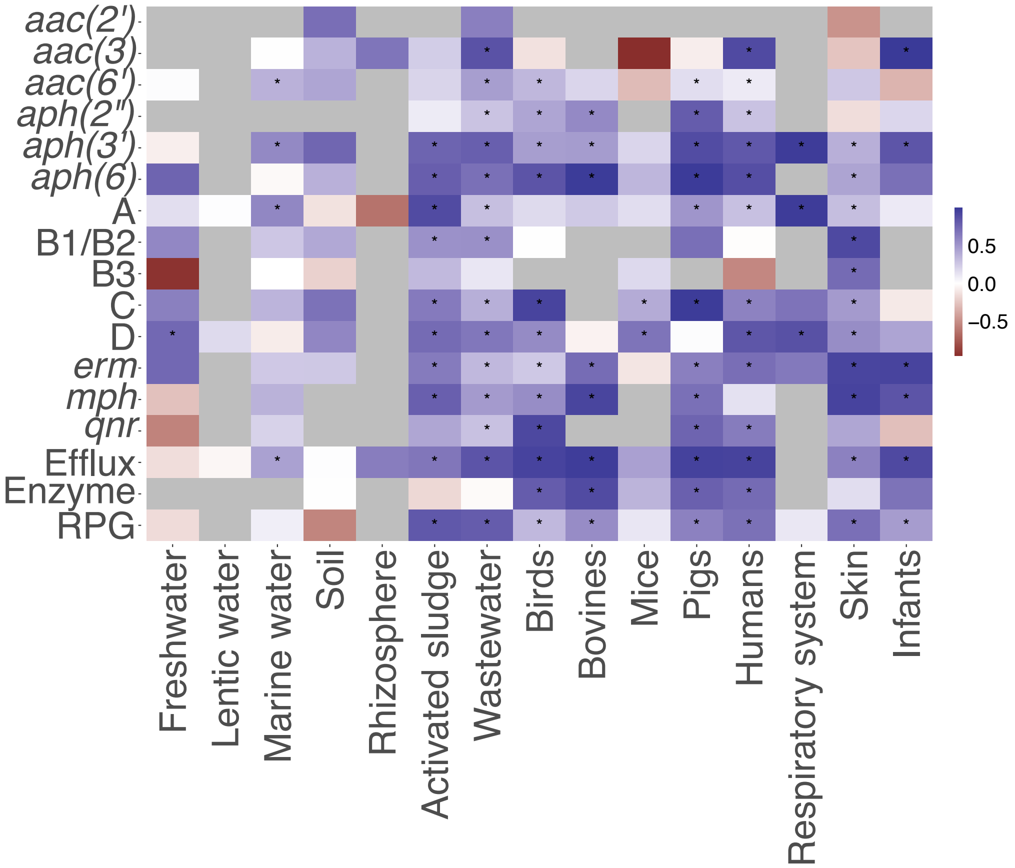

Figure S4: Pearson correlation between the latent and establish ARGs divided into gene class and environment. The labels Birds, Bovines, Mice, Pigs, Humans, and Infants denotes metagenomes from the corresponding digestive system. Respiratory system and skin only includes human samples. The color intensity reflect the size of the estimated correlation coefficient and a star (*) marks significant correlations (p<0.001). Gray squares indicate environment and gene classes with insufficient number of non-zero observations to calculate the correlation coefficient and/or the p-value.
